# Supplementary material for: Exploring the Aroma Fingerprint of Various Chinese Pear Cultivars through Qualitative and Quantitative Analysis of Volatile Compounds Using HS-SPME and GC×GC-TOFMS
Source: Molecules. 2023 Jun 15;28(12):4794. doi: 10.3390/molecules28124794 (PMC10301882; doi:10.3390/molecules28124794)
Supplement: Supplementary file 1 [file molecules-28-04794-s001.zip › Table S1.pdf]

Table S1 Aroma composition and content of different cultivars of *Pyrus ussuriensis* Maxim

No.	Volatile compounds	[1]R.T. (s)	[2]RI	Anli (ng/g)	Dongmili (ng/g)	Huagai (ng/g)	Jianbali (ng/g)	Jingbaili (ng/g)	Jinxiangshui (ng/g)	Nanguoli (ng/g)
Esters (102)										
1	Hexyl acetate	687, 1.280	1014.7	$476.81±102.83^{a}$	$120.98±16.47^{b}$	$100.87±3.50^{bc}$	[3]$37.25±6.57^{cd}$	$46.26±1.92^{cd}$	$26.02±3.92^{d}$	$3.05±0.56^{d}$
2	Ethyl acetate	132, 0.760	605.04	$180.96±5.45^{b}$	$148.09±29.27^{b}$	$40.06±5.56^{cd}$	$87.35±13.05^{c}$	$0.47±0.12^{d}$	$525.29±75.51^{a}$	$17.93±2.79^{d}$
3	Isoamyl acetate	435, 1.140	875.2	[4]-	-	-	$59.54±6.51^{a}$	-	-	-
4	Isobutyl acetate	276, 0.970	772.01	-	$36.25±7.26^{a}$	$8.80±1.23^{c}$	$21.18±4.21^{b}$	$4.35±0.90^{cd}$	$8.82±1.65^{c}$	$5.05±0.73^{cd}$
5	Butyl acetate	333, 1.100	814.49	$147.93±17.93^{c}$	$314.82±43.60^{b}$	$90.97±4.56^{cd}$	$84.99±7.98^{cd}$	$56.48±4.17^{d}$	$787.42±94.84^{a}$	$36.29±7.97^{d}$
6	2-Methylbutyl acetate	441, 1.120	878.76	-	$15.96±1.43^{b}$	$13.13±1.87^{c}$	$36.19±2.97^{a}$	$8.47±0.40^{d}$	-	$8.95±1.05^{d}$
7	Methyl 2-methyl-2-butenoate	417, 1.290	864.58	-	$15.38±1.34^{a}$	-	-	-	-	-
8	Ethyl caproate	663, 1.290	1001.8	$159.73±1.36^{b}$	$22.39±3.81^{d}$	$1.14±0.76^{d}$	$110.55±0.25^{c}$	$1.72±0.48^{d}$	$264.42±41.98^{a}$	$3.43±0.26^{d}$
9	Methyl caproate	522, 1.250	924.82	$153.20±27.75^{b}$	$66.50±7.17^{c}$	$23.91±0.71^{de}$	$23.29±7.10^{de}$	$60.00±14.80^{cd}$	$246.00±48.82^{a}$	$14.26±0.27^{e}$
10	Ethyl *(2Z)*-but-2-enoate	381, 1.280	843.15	$112.42±8.84^{a}$	$117.43±20.77^{a}$	$2.79±1.11^{c}$	$24.26±1.75^{b}$	-	$15.19±1.88^{bc}$	$13.67±3.18^{bc}$
11	*(Z)*-hex-2-enyl acetate	693, 1.360	1018	$12.42±0.73^{c}$	$36.21±4.85^{b}$	$107.80±11.87^{a}$	$15.35±2.25^{c}$	-	-	-
12	Methyl acetate	102, 0.620	500.37	$115.74±8.84^{b}$	$57.67±5.47^{cd}$	$25.52±4.50^{d}$	$25.92±1.95^{d}$	$56.73±3.85^{cd}$	273.74±57.42	$82.96±12.89^{bc}$

									^{a}	
13	Ethyl butyrate	312, 1.110	802	85.23±2.20^{b}	95.60±13.35^{b}	2.07±0.73^{d}	43.20±8.88^{c}	0.22±0.03^{d}	161.26±42.65^{a}	11.09±1.59^{d}
14	Methyl butyrate	213, 0.930	718.17	53.72±7.82^{b}	48.71±12.50^{b}	16.12±2.37^{c}	8.88±0.42^{c}	12.82±1.98^{c}	122.34±2.65^{a}	42.72±4.88^{b}
15	Ethyl caprylate	1020, 1.270	1198.5	23.05±0.57^{c}	2.44±0.27^{d}	0.24±0.04^{e}	33.54±2.32^{b}	0.42±0.07^{e}	2.35±0.34^{d}	43.40±0.41^{a}
16	Propyl acetate	204, 0.890	710.45	24.84±2.37^{c}	73.01±11.45^{a}	5.97±0.62^{d}	66.15±5.59^{a}	0.52±0.07^{d}	33.24±1.55^{b}	-
17	Ethyl propionate	201, 0.890	707.89	18.92±2.06^{b}	5.82±0.68^{d}	-	4.18±0.16^{e}	-	9.66±0.80^{c}	32.07±0.26^{a}
18	Methyl 2-ethylacrylate	417, 1.260	864.56	-	-	-	-	3.16±0.32^{a}	-	-
19	Ethyl 2-methyl butyrate	390, 1.070	848.38	19.38±0.64^{c}	27.06±2.00^{b}	0.05±0.03^{e}	55.81±3.47^{a}	0.04±0.00^{e}	6.93±1.36^{d}	60.64±6.06^{a}
20	Hexyl 2-Methyl butyrate	1086, 1.210	1237.6	-	-	0.01±0.00^{d}	1.28±0.13^{a}	0.05±0.00^{d}	0.60±0.20^{b}	0.23±0.01^{c}
21	Hex-2-en-1-ol acetate	693,1.370	1018	-	-	-	-	-	49.26±3.37^{a}	30.12±1.62^{b}
22	Ethyl 2-hexenoate	744, 1.420	1045.4	22.50±0.44^{a}	2.97±0.61^{d}	0.28±0.12^{e}	14.06±2.84^{b}	0.24±0.02^{e}	0.71±0.07^{de}	10.73±1.83^{c}
23	Methyl crotonate	261, 1.120	759.33	13.99±1.20^{b}	47.37±5.80^{a}	43.16±5.07^{a}	12.03±1.11^{bc}	9.41±0.64^{bc}	7.31±0.60^{cd}	2.81±0.15^{d}
24	Ethyl crotonate	381, 1.250	843.14	-	-	-	-	0.27±0.04^{a}	-	-
25	Ethyl *(E)*-hex-3-enoate	681, 1.330	1011.5	11.28±0.75^{a}	1.59±0.38^{d}	0.10±0.00^{e}	2.74±0.55^{c}	-	5.29±0.44^{b}	3.01±0.43^{c}
26	Ethyl *(E)*-2-octenoate	1101, 1.410	1246.7	9.02±1.47^{b}	-	-	7.23±0.22^{b}	-	0.76±0.03^{c}	15.77±3.37^{a}
27	Methyl octanoate	891, 1.280	1125.6	13.74±2.80^{c}	16.13±1.81^{c}	7.45±0.35^{d}	6.57±0.47^{d}	24.64±1.24^{b}	3.14±0.33^{d}	32.96±0.16^{a}
28	Methyl isobutyrate	177, 0.810	676.47	-	-	-	-	0.54±0.07^{a}	-	-
29	Ethyl isobutyrate	255, 0.920	754.03	3.46±0.27^{c}	7.55±1.23^{b}	-	16.95±0.56^{a}	-	1.85±0.15^{d}	3.99±0.74^{c}
30	*(E)*-2-hexenoic acid,	597, 1.370	965.86	4.50±0.79^{a}	1.95±0.10^{c}	3.00±0.30^{b}	3.12±0.17^{b}	1.28±0.24^{d}	0.92±0.06^{d}	1.32±0.08^{d}

	methyl ester									
31	Octyl acetate	1044, 1.260	1212.7	3.21±0.42[c]	5.72±0.66[b]	4.65±0.22[bc]	3.15±0.48[c]	0.33±0.14[d]	2.53±0.12[cd]	17.03±3.29[a]
32	*(E)*-3-hexenyl acetate	675, 1.290	1008.3	3.80±0.47[d]	12.82±0.89[b]	2.87±0.40[d]	13.85±0.42[b]	7.03±0.16[c]	23.19±2.31[a]	6.09±1.25[c]
33	*(E,Z)*-2,4-Decadienoic acid, ethyl ester	1452, 1.500	1470.3	2.70±0.53[a]	-	-	1.47±0.10[b]	-	-	3.39±1.36[a]
34	Heptyl acetate	870, 1.240	1113.7	3.04±0.22[c]	270.06±12.56[a]	12.19±2.50[bc]	5.82±0.53[c]	4.62±0.23[c]	9.97±0.67[bc]	15.58±0.41[b]
35	Ethyl methacrylate	291, 1.040	784.88	-	-	-	4.77±0.27[a]	-	-	-
36	Ethyl acrylate	189, 0.900	695.64	-	-	-	1.69±0.24[a]	-	-	0.55±0.03[b]
37	Diethyl carbonate	291, 1.150	784.97	2.49±0.24[b]	-	-	4.81±0.25[a]	-	-	-
38	Ethyl heptanoate	843, 1.250	1098.6	3.48±0.37[b]	0.74±0.26[d]	-	2.77±0.19[c]	-	0.77±0.04[d]	8.79±0.13[a]
39	Pentyl acetate	504, 1.160	914.94	2.84±0.14[e]	9.86±0.49[c]	4.39±0.52[de]	4.37±0.38[de]	5.70±0.69[d]	22.36±2.59[a]	18.59±1.53[b]
40	Decyl acetate	1365, 1.270	1412.2	-	0.83±0.11[a]	0.07±0.02[c]	-	-	-	0.16±0.06[b]
41	Pentyl acetate	480, 1.150	901.82	3.27±0.63[b]	0.64±0.02[c]	-	0.33±0.01[c]	-	3.75±0.38[b]	8.06±0.82[a]
42	Ethyl tiglate	549, 1.300	939.6	2.76±0.28[c]	32.76±6.28[a]	1.13±0.19[c]	27.78±2.25[b]	0.09±0.02[c]	0.40±0.14[c]	0.56±0.04[c]
43	Propyl *(E)*-2-methylbut-2-enoate	729, 1.340	1037.3	-	0.21±0.03[a]	-	-	-	-	-
44	Methyl tiglate	417, 1.290	864.58	-	-	3.45±0.21[a]	-	-	-	-
45	Ethyl *(Z)*-hex-3-enoate	672, 1.310	1006.7	2.14±0.23[a]	-	-	-	-	-	-
46	Ethyl phenylacetate	1101, 1.910	1247	1.74±0.13[a]	-	-	0.48±0.01[c]	-	-	0.69±0.14[b]
47	Methyl propionate	141, 0.750	619.3	1.58±0.08[d]	5.97±0.61[b]	1.68±0.35[d]	1.78±0.22[d]	5.40±0.67[b]	8.93±0.65[a]	2.86±0.28[c]

48	Methyl *(E)*-2-octenoate	972, 1.420	1171.5	2.04±0.03^{a}	0.33±0.05^{de}	0.36±0.03^{cd}	0.32±0.02^{de}	0.48±0.16^{c}	0.21±0.08^{e}	0.82±0.02^{b}
49	2-Buten-1-ol, acetate	351, 1.190	825.25	-	0.44±0.04^{b}	1.82±0.52^{a}	0.55±0.02^{b}	-	-	-
50	Methyl heptanoate	708, 1.230	1026	2.89±0.29^{bc}	3.32±0.38^{b}	0.65±0.12^{d}	0.95±0.04^{d}	2.88±0.08^{bc}	2.59±0.61^{c}	4.40±0.16^{a}
51	Methyl 2-methylbutyrate	279, 0.960	774.56	1.42±0.21^{e}	7.00±0.74^{b}	2.58±0.38^{d}	14.56±0.86^{a}	1.57±0.05^{e}	6.80±0.90^{b}	4.14±0.19^{c}
52	Propyl 2-Methylbutyrate	561, 1.130	946.06	-	-	-	0.35±0.03^{a}	-	-	0.18±0.03^{b}
53	*(E,Z)*-2,4-Decadienoic acid, methyl ester	1341, 1.520	1396.5	0.80±0.04^{b}	0.33±0.03^{cd}	0.17±0.01^{de}	0.41±0.01^{c}	1.40±0.25^{a}	-	1.37±0.13^{a}
54	Methyl valerate	348, 1.090	823.41	1.10±0.08^{c}	0.90±0.07^{c}	0.28±0.07^{d}	-	-	6.68±0.07^{a}	3.72±0.64^{b}
55	Methyl *(3E)*-3-hexenoate	537, 1.280	933.03	0.82±0.03^{b}	1.04±0.18^{a}	0.64±0.02^{c}	-	0.17±0.00^{e}	0.16±0.01^{e}	0.49±0.03^{d}
56	Phenethyl acetate	1122, 1.890	1259.5	0.45±0.09^{c}	0.84±0.02^{a}	0.12±0.01^{d}	0.51±0.04^{cb}	0.11±0.00^{d}	0.18±0.00^{d}	0.54±0.05^{b}
57	Hexyl butyrate	1008, 1.250	1191.7	0.63±0.01^{d}	0.56±0.02^{d}	0.22±0.04^{e}	0.50±0.01^{d}	1.52±0.07^{c}	3.86±0.11^{b}	5.19±0.15^{a}
58	Butyl butyrate	654,1.190	996.9	-	-	-	-	-	0.42±0.04^{a}	0.14±0.04^{b}
59	Ethyl benzoate	975, 1.790	1173.4	0.34±0.03^{b}	0.74±0.03^{a}	-	-	-	-	0.22±0.02^{c}
60	Methyl benzoate	840, 1.840	1097.3	-	-	0.05±0.00^{c}	-	-	0.60±0.01^{a}	0.17±0.02^{b}
61	Dimethyl carbonate	135, 0.790	609.85	-	-	-	-	1.22±0.14^{a}	-	-
62	*(Z)*-4-Octenoic acid, ethyl ester	1005, 1.340	1190	0.32±0.02^{c}	-	-	0.60±0.03^{b}	-	0.08±0.01^{cd}	1.65±0.26^{a}
63	*(Z)*-4-Decenoic acid, methyl ester	1206, 1.370	1309.9	-	-	-	0.05±0.01^{c}	0.12±0.01^{b}	-	0.49±0.07^{a}
64	*(E)*-2-Heptenoic acid, ethyl ester	930, 1.400	1147.7	-	-	-	0.04±0.00^{b}	-	-	0.09±0.00^{a}

65	Ethyl *(E)*-pent-2-enoate	567, 1.350	949.46	0.43±0.01[a]	-	-	-	-	-	0.09±0.00[b]
66	Ethyl decanoate	1341, 1.270	1396.3	0.17±0.00[c]	-	-	0.40±0.08[b]	-	-	0.84±0.01[a]
67	Methyl decanoate	1230, 1.290	1325.2	-	-	0.16±0.01[b]	0.13±0.02[c]	0.44±0.04[a]	-	-
68	4-Octenoic acid, methyl ester	876, 1.340	1117.2	0.26±0.01[c]	-	0.40±0.03[b]	0.20±0.02[d]	0.28±0.00[c]	0.10±0.00[e]	0.61±0.05[a]
69	*(Z)*-3-Octenoic acid, ethyl ester	1026, 1.350	1202	-	-	-	0.16±0.00[b]	-	-	0.63±0.03[a]
70	Propyl butyrate	474, 1.130	898.4	0.23±0.02[c]	0.24±0.03[c]	0.03±0.00[d]	-	-	0.84 ±0.07[b]	1.00±0.04[a]
71	Propyl hexanoate	837, 1.230	1095.3	0.14±0.02[c]	-	-	-	-	0.22±0.01[b]	1.16±0.12[a]
72	1-Methylethyl hexanoate	732,1.130	1038.8	-	-	-	-	-	0.56±0.10[a]	0.12±0.02[b]
73	2-Methylpropyl hexanoate	936,1.200	1151	-	-	-	-	-	-	0.06±0.00[a]
74	Hexyl hexanoate	1326, 1.270	1386.7	-	-	0.04±0.00[c]	0.29±0.02[c]	0.67±0.10[b]	0.73±0.03[b]	2.78±0.11[a]
75	Hex-5-enoic acid, ethyl ester	669,1.310	1005.1	-	-	-	-	-	-	0.10±0.00[a]
76	Hexyl pentanoate	1173,1.260	1289.5	-	-	-	-	-	-	0.04±0.00[a]
77	Hexyl caprylate	1614,1.280	1583.2	-	-	-	-	-	-	0.01±0.00[a]
78	2-Pentenoic acid, ethyl	567, 1.340	949.45	-	-	-	0.17±0.01[a]	-	-	-

	ester									
79	Butanoic acid, 2-methyl-, 2-methylbutyl ester	855, 1.150	1105.2	-	-	-	0.13±0.01[a]	-	-	-
80	Butanoic acid, 2-methylpropyl ester	579,1.130	955.89	-	-	-	-	-	-	0.10±0.00[a]
81	Dodecanoic acid, ethyl ester	1632,1.27 0	1595.9	-	-	-	-	-	-	0.03±0.00[a]
82	*(Z)*-3-Octen-1-ol, acetate	1020, 1.340	1198.5	-	0.35±0.03[a]	0.23±0.03[b]	-	-	-	-
83	*(Z)*-3-Octenoic acid, methyl ester	900, 1.380	1130.8	-	-	0.03±0.00[b]	-	-	-	0.07±0.00[a]
84	*(Z)*-4-Decenoic acid, ethyl ester	1317,1.34 0	1381	-	-	-	-	-	-	0.07±0.00[a]
85	5-Hexenoic acid, methyl ester	498,1.260	911.71	-	-	-	-	-	-	0.03±0.00[a]
86	4-Decenoic acid, methyl ester	1206, 1.370	1309.9	-	0.27±0.01[a]	-	-	-	-	-
87	4-Hexen-1-ol, acetate	1038,1.36 0	1209.2	-	-	-	-	-	0.10±0.00[a]	-
88	Methyl *(Z)*-hex-3-enoate	537, 1.290	933.03	-	0.26±0.10[a]	-	0.06±0.01[b]	-	-	-
89	Ethyl 3-(methylthio)-*(E)*-2-propenoate	990, 1.980	1181.9	-	0.22±0.05[a]	-	-	-	-	0.24±0.03[a]
90	Methyl 3-(methylthio)	846, 2.040	1100.6	-	-	0.24±0.00[a]	-	-	-	-

	-*(E)*-2-propenoate									
91	3-(Methylthio) propanoic acid ethyl ester	849,1.730	1102.2	-	-	-	-	-	-	0.71±0.03[a]
92	Methyl 3-methylthiopropionate	708,1.810	1026.3	-	-	-	-	-	-	0.23±0.03[a]
93	3-Methyl-3-buten-1-ol, acetate	450, 1.210	884.17	-	1.20±0.02[a]	-	-	-	-	-
94	2-Methylbut-2-en-1-yl acetate	519, 1.300	923.21	-	-	1.74±0.06[a]	-	-	-	-
95	γ-Butyrolactone	504, 2.680	915.77	-	-	-	1.30±0.31[a]	-	-	-
96	Prenylacetate	519, 1.280	923.2	-	-	-	-	0.28±0.02[a]	-	-
97	*(Z)*-3-Decen-1-yl acetate	1332, 1.350	1390.6	-	1.06±0.18[a]	-	-	-	-	-
98	Hexyl tiglate	1242, 1.380	1332.9	-	-	0.03±0.00[a]	-	-	-	-
99	Propanoic acid, 2-methyl-, 3-hydroxy-2,2,4-trimethy lpentyl ester	1311, 1.460	1377.2	-	-	0.37±0.05[a]	-	-	-	-
100	Diisobutyl phthalate	1989, 1.950	1875.7	-	-	0.26±0.04[b]	-	-	-	1.18±0.02[a]
101	Butanoic acid, 2-methyl-, 2-methylpropyl ester	666, 1.130	1003.4	-	-	-	0.27±0.02[a]	-	-	-

102	Hexyl propionate	858,1.230	1107	-	-	-	-	-	1.24±0.13[a]	0.79±0.06[b]
	Subtotal			1707.93	1640.59	534.29	896.63	322.40	2682.19	564.74
Alcohols (14)										
1	Ethanol	90, 0.600	455.95	36.91±0.81[b]	25.09±1.16[c]	2.47±0.23[e]	50.65±8.03[a]	14.28±1.14[d]	29.91±4.18[c]	36.93±1.25[b]
2	1-Hexanol	420, 1.190	866.31	31.39±1.24[b]	59.71±12.59[a]	24.90±4.04[b]	75.39±0.13[a]	77.23±21.96[a]	61.26±13.81[a]	11.40±2.62[b]
3	*(Z)*-2-Hexen-1-ol	417, 1.200	864.53	0.75±0.04[b]	-	-	-	-	5.52±1.63[a]	-
4	*(Z)*-4-Decen-1-ol	1119, 1.390	1257.4	-	-	0.02±0.00[b]	-	0.05±0.00[a]	-	0.05±0.01[a]
5	1-Octanol	792, 1.250	1071.2	0.25±0.03[de]	5.56±0.60[a]	2.36±0.29[b]	1.23±0.15[c]	0.16±0.02[e]	0.14±0.03[e]	0.71±0.16[d]
6	*n*-Heptanol	606, 1.220	970.69	-	0.77±0.12[a]	0.15±0.01[bc]	0.21±0.01[b]	0.08±0.01[cd]	0.14±0.04[bc]	0.09±0.01[c]
7	1-Nonanol	972,1.280	1171.4	-	-	-	-	-	-	0.02±0.01[a]
8	*(E)*-2-Hexen-1-ol	417, 1.220	864.54	-	-	1.48±0.27[c]	3.89±0.66[b]	6.91±1.09[a]	-	-
9	2-Nonanol	846, 1.190	1100.2	-	-	0.10±0.02[b]	-	-	-	0.36±0.07[a]
10	3-Octanol	654, 1.150	996.88	-	-	0.03±0.01[a]	-	-	-	-
11	3,7,11-Trimethyl-6,10-dodecadien-1-ol	1764, 1.450	1693.6	-	-	0.03±0.01[a]	-	-	-	-
12	6-Methyl-5-Hepten-2-ol	648, 1.230	993.64	-	-	0.02±0.00[a]	-	-	-	0.02±0.00[a]
13	(±)-2-Methyl-1-butanol	228, 0.920	730.97	-	-	-	0.78±0.06[a]	-	-	-
14	4-Methyl-1-pentanol	423, 1.210	868.1	-	-	-	-	26.08±1.53[a]	-	-
	Subtotal			69.3	91.13	31.56	132.15	124.79	96.97	49.58
Alkenes (18)										

1	*(Z,E)*-*α*-Farnesene	1491, 1.290	1496.2	1.65±0.00[c]	0.35±0.11[d]	0.24±0.01[d]	2.85±0.24[b]	3.03±0.59[b]	0.12±0.02[d]	5.98±1.32[a]
2	*(Z,Z)*-*α*-Farnesene	1539,1.280	1530	-	-	-	-	-	-	0.25±0.04[a]
3	*(E)*-*β*-Farnesene	1437, 1.230	1460.1	2.91±0.64[a]	-	0.14±0.02[bc]	0.40±0.08[bc]	0.48±0.10[bc]	-	0.51±0.14[b]
4	*α*-Farnesene	1512, 1.310	1510.8	71.95±12.64[a]	2.68±0.26[c]	5.63±0.93[c]	48.45±9.63[b]	66.11±17.61[a]	2.52±0.80[c]	14.08±0.92[c]
5	2,3-Dimethyl-1,3-Butadiene	138, 0.660	614.4	-	1.09±0.28[b]	0.13±0.03[c]	-	-	2.55±0.19[a]	-
6	1,3,5,7-Cyclooctatetraene	459, 1.360	889.61	0.20±0.01[a]	-	0.13±0.08[b]	-	0.08±0.01[b]	-	-
7	1,3,5-Cycloheptatriene	264, 1.030	761.81	-	-	-	1.02±0.11[a]	0.89 ±0.19[a]	-	0.64±0.03[b]
8	Limonene	714, 1.140	1029.2	-	-	0.04±0.01[b]	-	-	-	0.19±0.02[a]
9	3-(4-methylpent-3-enyl) Furan	849, 1.280	1101.9	-	-	0.02±0.00[a]	-	-	-	-
10	*(E)*-1,4-Hexadiene	141, 0.650	619.14	-	-	-	-	1.05±0.25[a]	-	-
11	*β*-Myrcene	645,1.060	991.91	-	-	-	-	-	-	0.04±0.00[a]
12	*(Z,Z)*-2,4-Hexadiene	141, 0.650	619.14	-	-	-	0.76±0.08[b]	1.19±0.03[a]	-	0.30±0.03[c]
13	*(Z)*-1,3-Pentadiene	105, 0.560	511.23	-	-	-	0.53±0.02[a]	-	-	-
14	*(E)*-3-Hexene	129, 0.630	600.08	-	-	-	-	0.22±0.10[a]	-	-
15	*α*-Curcumene	1479,1.420	1488.3	-	-	-	-	-	0.03±0.00[a]	-
16	*(E)*-*β*-Ocimene	753,1.130	1050.1	-	-	-	-	-	-	0.03±0.00[a]

17	*(3E,5E)*-1,3,5-Heptatriene	273, 0.930	769.41	-	-	-	0.83±0.11[a]	0.61±0.13[b]	-	-
18	*(E)*-1-Methyl-4-(6-methylhept-5-en-2-ylidene) cyclohex-1-ene	1602, 1.360	1574.7	-	-	-	-	0.46±0.09[a]	-	-
	Subtotal			76.71	4.12	6.33	54.84	73.84	5.22	22.02
Aldehydes (20)										
1	*(E)*-2-methyl-2-butenal	237, 1.100	738.81	-	0.42±0.11[b]	-	1.02±0.13[a]	0.46±0.05[b]	-	-
2	Hexanal	309, 1.170	800.25	15.23±2.90[d]	42.07±3.83[a]	24.86±4.29[c]	32.93±1.50[b]	26.57±2.21[c]	16.01±2.17[d]	3.03±0.04[e]
3	Nonanal	852, 1.320	1103.6	0.63±0.19[a]	-	0.12±0.01[b]	0.62±0.06[a]	0.53±0.14[a]	-	-
4	Octanal	666, 1.290	1003.5	-	0.94±0.10[a]	0.35±0.04[b]	-	0.11±0.01[c]	-	-
5	Heptanal	477, 1.220	900.22	-	-	0.04±0.01[b]	0.03±0.01[bc]	0.09±0.05[a]	-	-
6	Decanal	1032, 1.330	1205.6	-	-	-	0.08±0.01[b]	0.18±0.02[a]	0.06±0.01[bc]	0.05±0.01[c]
7	*(Z)*-3-Hexenal	306, 1.170	797.8	-	-	-	0.29±0.04[a]	-	-	-
8	*(E)*-2-Heptenal	579, 1.430	956.06	-	-	-	0.17±0.01[a]	-	-	-
9	*(Z)*-2-Butenal	153, 0.920	638.59	-	-	-	0.52±0.16[b]	1.25±0.24[a]	-	-
10	Croton aldehyde	156, 0.910	643.33	-	-	-	-	0.08±0.01[a]	-	-
11	*(E,E)*-2,4-Hexadienal	495, 1.620	910.27	-	0.17±0.04[c]	0.15±0.02[c]	0.68±0.05[a]	0.27±0.05[c]	0.41±0.16[b]	-
12	2-Hexenal	384, 1.310	844.96	-	-	-	-	0.19±0.08[b]	-	2.11±0.31[a]
13	2-Methylbut-2-enal	237,1.120	738.83	-	-	-	-	-	-	0.15±0.04[a]
14	2 - Methyl propenal	117,0.680	556.02	-	-	-	-	-	-	0.26±0.03[a]

15	*(E)*-2-Octenal	768, 1.450	1058.4	$0.09±0.01^{a}$	-	$0.03±0.01^{b}$	$0.09±0.03^{a}$	$0.06±0.02^{a}$	-	$0.03±0.01^{b}$
16	*(E)*-2-Nonenal	903, 1.220	1132.4	-	-	$0.02±0.01^{b}$	-	-	-	$0.07±0.02^{a}$
17	*(E)*-2-Hexenal	393, 1.370	850.35	$4.42±0.21^{cd}$	$16.66±3.13^{b}$	$7.40±0.31^{c}$	$22.20±0.69^{a}$	$13.07±4.27^{b}$	$20.51±1.14^{a}$	$2.60±1.15^{d}$
18	*(E)*-2,4-Decadienal	1182, 1.550	1295	-	-	-	-	$0.07±0.01^{a}$	-	-
19	Benzeneacetaldehyde	744, 2.060	1045.8	-	-	$0.14±0.09^{b}$	$0.44±0.13^{a}$	-	-	$0.08±0.03^{bc}$
20	Benzaldehyde	588, 1.910	961.23	-	-	$0.06±0.04^{b}$	$0.12±0.02^{a}$	$0.07±0.01^{b}$	-	$0.02±0.01^{c}$
	Subtotal			20.37	60.26	33.17	59.19	43	36.99	8.4
Ketones (5)										
1	Methyl heptenone	639, 1.380	988.81	$1.16±0.24^{b}$	-	$0.36±0.04^{c}$	$1.68±0.28^{a}$	$1.42±0.31^{ab}$	$0.36±0.10^{c}$	$1.29±0.26^{ab}$
2	Geranyl acetone	1431, 1.500	1456.3	-	-	$0.15±0.03^{a}$	-	-	-	$0.18±0.05^{a}$
3	2,3-Octanedione	633, 1.280	985.47	-	-	$0.15±0.02^{a}$	-	-	-	-
4	2,3-Butanedione	123, 0.720	578.34	-	-	-	$0.54±0.02^{a}$	-	-	-
5	Butenone	123, 0.720	578.34	-	-	-	-	$0.50±0.04^{a}$	-	$0.48±0.07^{a}$
	Subtotal			1.16	0	0.66	2.22	1.92	0.36	1.95
Alkanes (8)										
1	Tetradecane	1347, 0.980	1400	$1.40±0.05^{bc}$	$0.19±0.01^{e}$	$0.37±0.02^{e}$	$1.25±0.05^{c}$	$1.82±0.07^{a}$	$0.69±0.07^{d}$	$1.52±0.29^{b}$
2	Hexadecane	1638, 1.010	1600	$0.43±0.07^{a}$	-	-	-	-	-	-

3	Tridecane	1191, 0.950	1300	0.41±0.08^{bc}	0.15±0.01^{d}	0.15±0.02^{d}	0.49±0.15^{ab}	0.32±0.08^{c}	0.34±0.03^{c}	0.57±0.01^{a}
4	3-Methyl-tridecane	1302, 0.970	1371.1	-	-	0.03±0.00^{b}	0.04±0.01^{a}	-	-	-
5	Undecane	846, 0.890	1100	-	0.23±0.03^{a}	0.07±0.00^{c}	0.14±0.03^{b}	0.10±0.01^{bc}	0.13±0.03^{b}	-
6	Heptadecane	1773, 1.020	1700	-	-	0.04±0.00^{b}	-	-	-	0.18±0.12^{a}
7	2,6,10-Trimethyl-Dodecane	1311, 0.940	1376.9	-	-	0.03±0.00^{b}	-	-	-	0.07±0.00^{a}
8	Decane	660, 0.870	1000	-	-	0.02±0.00^{a}	-	-	-	-
	Subtotal			2.24	0.57	0.71	1.92	2.24	1.16	2.34
Others (7)										
1	*o*-Xylene	429, 1.180	871.65	-	2.79±0.12^{a}	1.33±0.03^{b}	-	-	2.70±0.26^{a}	-
2	*p*-Xylene	426, 1.180	869.87	-	-	-	10.35±0.13^{a}	-	-	8.55±0.56^{b}
3	1,2,3-Trimethyl-Benzene	648, 1.340	993.7	-	-	0.19±0.02^{a}	-	-	-	-
4	4-Ethyl-1,2-dimethyl-Benzene	819,1.370	1085.7	-	-	-	-	-	-	0.09±0.01^{a}
5	1-Ethyl-4-methylbenzene	591, 1.270	962.52	-	-	-	0.77±0.04^{a}	0.65±0.03^{b}	-	-
6	*o*-Cymene	804, 1.350	1077.7	-	-	0.06±0.00^{a}	-	-	-	-
7	Denderalasin	1608, 1.410	1579	-	-	0.23±0.01^{a}	-	-	-	-
	Subtotal			0	2.79	1.81	11.12	0.65	2.7	8.64

Total	1877.71	1799.46	608.53	1158.07	568.84	2825.59	657.67

[1]R.T. refers to the retention time. [2]RI refers to the calculated retention index. [3]Data are means ± SD of three replications. [4]"-" represents no data.
