# Supplementary material for: Exploring the Aroma Fingerprint of Various Chinese Pear Cultivars through Qualitative and Quantitative Analysis of Volatile Compounds Using HS-SPME and GC×GC-TOFMS
Source: Molecules. 2023 Jun 15;28(12):4794. doi: 10.3390/molecules28124794 (PMC10301882; doi:10.3390/molecules28124794)
Supplement: Supplementary file 1 [file molecules-28-04794-s001.zip › Table S2.pdf]

Table S2 "ABC" value of scent of aroma substances in different cultivars of *Pyrus ussuriensis* Maxim

Components name	Relative impact value	A	B	C	D	E	F	G	H	I	J	K	L	M	N	O	P	Q	R	S	T	U	V	W	X	Y	Z
Hexyl acetate	150	10	-	-	10	-	60	10	10	-	-	-	-	-	-	-	-	-	-	-	-	-	-	-	-	-	-
Ethyl acetate	50	-	-	-	20	-	40	-	-	-	-	-	-	-	-	-	-	-	-	-	-	-	-	-	-	-	40
Isoamyl acetate	800	-	-	-	5	-	95	-	-	-	-	-	-	-	-	-	-	-	-	-	-	-	-	-	-	-	-
Isobutyl acetate	80	-	-	-	20	-	70	-	-	-	-	-	-	-	-	-	-	-	-	-	-	-	-	-	-	-	10
Butyl acetate	80	-	-	-	10		90	-	-	-	-	-	-	-	-	-	-	-	-	-	-	-	-	-	-	-	-
Ethyl caproate	100	-	-	-	20	-	40	30	-	-	-	-	-	-	10	-	-	-	-	-	-	-	-	-	-	-	-
Methyl caproate	100	-	-	-	10	10	60	20	-	-	-	-	-	-	-	-	-	-	-	-	-	-	-	-	-	-	-
Methyl acetate	70	-	-	-	10	10	-	30	-	-	-	-	-	-	-	-	-	-	-	-	-	-	-	-	-	-	50
Ethyl butyrate	200	-	10	-	-	-	90	-	-	-	-	-	-	-	-	-	-	-	-	-	-	-	-	-	-	-	-
Ethyl caprylate	125	40	-	-	10	-	30	-	-	-	-	-	-	-	-	-	-	-	-	-	-	1<br>0	-	-	-	-	10
propyl acetate	80	-	-	-	10	-	80	-	-	-	-	-	-	-	-	-	-	-	-	-	-	-	-	-	-	-	10
Ethyl 2-methyl butyrate	280	-	10	-	-	-	90	-	-	-	-	-	-	-	-	-	-	-	-	-	-	-	-	-	-	-	-
Octyl acetate	200	20	-	-	10	-	60	-	-	-	-	-	-	-	-	10	-	-	-	-	-	-	-	-	-	-	-
(Z)-3-hexenyl acetate	800	-	-	-	-	-	64	36	-	-	-	-	-	-	-	-	-	-	-	-	-	-	-	-	-	-	-
(E,Z)-2, 4-Decadienoic	330	30	-	-	-	-	50	20	-	-	-	-	-	-	-	-	-	-	-	-	-	-	-	-	-	-	-

acid, ethyl ester																											
Heptyl acetate	300	50	-	-	-	-	40	10	-	-	-	-	-	-	-	-	-	-	-	-	-	-	-	-	-	-	-
Ethyl heptanoate	150	-	-	-	-	-	81	-	-	-	-	-	-	-	-	-	-	-	-	-	-	-	-	-	-	19	-
Pentyl acetate	100	-	-	-	20	-	80	-	-	-	-	-	-	-	-	-	-	-	-	-	-	-	-	-	-	-	-
Ethyl phenylacetate	65	-	-	-	-	-	20	-	-	-	-	-	-	-	-	-	60	-	-	-	-	-	20	-	-	-	-
(E,Z)-2, 4-Decadienoic acid, methyl ester	260	40	-	20	-	-	40	-	-	-	-	-	-	-	-	-	-	-	-	-	-	-	-	-	-	-	-
Phenethyl acetate	90	-	-	-	-	-	10	-	-	-	-	-	-	-	-	-	5	-	85	-	-	-	-	-	-	-	-
Butyl butyrate	140	-	-	-	-	-	73	27	-	-	-	-	-	-	-	-	-	-	-	-	-	-	-	-	-	-	-
Ethyl benzoate	70	-	-	-	-	-	40	-	-	-	-	-	-	-	60	-	-	-	-	-	-	-	-	-	-	-	-
Methyl benzoate	500	-	-	-	-	-	19	-	-	-	-	-	-	-	76	5	-	-	-	-	-	-	-	-	-	-	-
Methyl decanoate	160	10	-	-	10	-	65	10	-	-	-	-	-	-	-	-	-	-	-	-	-	5	-	-	-	-	-
Hexyl hexanoate	100	20	-	-	10	-	60	-	10	-	-	-	-	-	-	-	-	-	-	-	-	-	-	-	-	-	-
Butanoic acid, 2-methyl-, 2-methylbutyl ester	400	-	-	-	10	-	80	-	-	-	-	-	-	-	-	-	-	-	-	-	-	-	-	-	-	-	10
Prenylacetate	500	-	-	-	-	-	71	29	-	-	-	-	-	-	-	-	-	-	-	-	-	-	-	-	-	-	-

Diisobutyl phthalate	10	-	-	-	-	-	-	-	10	-	-	-	-	10		10		20	30	-	-	-	-	-	-	20	-
Hexyl propionate	100	10	-	-	20	-	40	10	-	-	-	-	-	-	-	10			10	-	-	-	-	-	-	-	-
1-Hexanol	100	20	-	-	-	-	70	10	-	-	-	-	-	-	-	-	-	-		-	-	-	-	-	-	-	-
1-Octanol	100	70	-	-	15	-	15	-	-	-	-	-	-	-	-	-	-	-		-	-	-	-	-	-	-	-
n-Heptanol	120	10	-	-	-	10	10	10	-	-	-	-	10	-	-	-	-	-	50	-	-	-	-	-	-	-	-
1-Nonanol	100	75	-	20	5	-	-	-	-	-	-	-	-	-	-	-	-	-		-	-	-	-	-	-	-	-
(E)-2-Hexen-1-ol	1000	-	-	-	-	20	5	70	-	-	-	-	-	-	-	-	-	-	-	-	-	-	5	-	-	-	-
(3Z,6E)-3,7,11-trimethyldodeca-1,3,6,10-tetraene	180	10	-	-	-	-	-	10	10	-	-	30	-	-	-	10	-	-	-	-	-	-	-	30	-	-	-
(E)-*β*-Farnesene	180	10	-	-	-	-	-	10	10	-	-	30	-	-	-	10	-	-	-	-	-	-	-	30	-	-	-
Limonene	120	-	-	80	-	-	20	-	-	-	-	-	-	-	-	-	-	-	-	-	-	-	-	-	-	-	-
β-Myrcene	250	-	-	30	-	-	-	-	30	-	-	20	-	-	-	-	-	20	-	-	-	-	-	-	-	-	-
(E)-*β*-Ocimene	500	-	-	-	-	-	-	-	60	-	-	-	-	-	-	-	8	-	-	32	-	-	-	-	-	-	-
Hexanal	700	60	-	-	-	-	20	10	-	-	-	-	-	-	-	-	-	-	-	-	-	-	-	10	-	-	-
Nonanal	550	60	-	10	-	-	-	10	-	10	-	-	-	-	-	-	-		10		-	-	-	-	-	-	-
Octanal	1000	60	-	35	-	-	-	5	-	-	-	-	-	-	-	-	-	-	-	-	-	-	-	-	-	-	-
Heptanal	700	75	-	10	-	-	-	10	5	-	-	-	-	-	-	-	-	-	-	-	-	-	-	-	-	-	-
Decanal	1000	80	-	10	-	-	-	-	-	-	-	-	-	-	-	-	-	-	10	-	-	-	-	-	-	-	-
(E)-2-Nonenal	800	80	-	-	20	-	-	-	-	-	-	-	-	-	-	-	-	-	-	-	-	-	-	-	-	-	-
Benzeneacetalde	400	-	-	-	-	-	-	50	-	-	-	-	-	-	20	-	-	-	-	-	-	-	-	30	-	-	-

hyde																											
Benzaldehyde	500	-	-	-	-	-	50	-	-	-	-	-	-	-	50	-	-	-	-	-	-	-	-	-	-	-	-
Geranyl acetone	200	-	-	-	10	-	-	-	-	-	-	-	-	5	-	5	-		80		-	-	-	-	-	-	-
2,3-Butanedione	3000	-	-	-	75		25	-	-	-	-	-	-	-	-	-	-	-	-	-	-	-	-	-	-	-	-
