# Supplementary material for: Exploring the Aroma Fingerprint of Various Chinese Pear Cultivars through Qualitative and Quantitative Analysis of Volatile Compounds Using HS-SPME and GC×GC-TOFMS
Source: Molecules. 2023 Jun 15;28(12):4794. doi: 10.3390/molecules28124794 (PMC10301882; doi:10.3390/molecules28124794)
Supplement: Supplementary file 1 [file molecules-28-04794-s001.zip › Table S3.pdf]

Table S3 Aroma value of different cultivars of *Pyrus ussuriensis* Maxim

Aroma types	Anli	Dongmili	Huagai	Jianbali	Jingbaili	Jinxiangshui	Nanguoli
A	917861.35±147128.64	6006710.18±375645.36	229751.84±42714.78	1760711.17±92523.64	1326204.22±131822.10	905454.08±116593.39	732088.62±47090.15
B	54264.00±1797.31	75773.51±5587.15	137.01±86.14	156261.92±9709.28	121.11±13.75	19403.99±3798.62	170837.08±17053.30
C	7640.60±1215.66	34508.52±3727.26	14598.17±1902.61	6991.46±595.12	17273.67±2986.94	949.49±171.23	10694.67±1144.17
D	713312.23±52884.74	623973.41±77833.78	158447.52±13697.65	830619.23±61897.56	169876.99±25207.61	1693228.17±258905.91	247983.10±28791.69
E	234218.00±33944.96	107795.61±11144.50	71628.36±9255.07	119458.69±21706.67	237928.34±39391.96	437786.67±89054.18	72463.27±9312.62
F	20498269.76±2840197.99	22849652.84±2174243.98	4521531.23±363710.40	13292619.10±1539003.35	3006795.19±305300.82	24920107.24±4379968.95	4001065.52±488216.76
G	1279813.16±117251.54	3657290.07±128255.51	507916.69±83468.83	1367121.60±92695.17	1142459.67±138465.71	2673102.13±428130.80	493499.72±78293.70
H	8195.26±1163.33	28426.27±195.78	878.16±96.67	6230.71±633.11	7287.76±1521.60	953.82±72.21	13739.96±3126.89
I	0.00	0.00	0.00	0.00	0.00	0.00	9.08±0.56
J	0.00	0.00	0.00	0.00	0.00	0.00	0.00
K	24585.77±3489.98	1878.80±587.34	2025.41±151.43	17505.74±1772.47	18957.65±3745.59	664.60±130.66	35050.72±7904.19
L	81605.94±3221.52	156180.52±32879.99	64916.56±10520.70	196270.90±13347.86	200893.64±57107.38	159448.09±35948.37	29760.91±6837.68
M	7194.03±522.94	0.00	179.55±36.31	1967.67±54.59	0.00	0.00	3173.39±642.41
N	161159.79±1479.13	25484.11±3934.16	5758.30±2653.05	117113.25±1659.66	3524.15±785.25	287386.15±42327.91	12187.46±1450.98
O	14615.26±2002.05	39872.83±1525.11	10286.61±544.66	5835.25±590.82	6976.13±1519.28	8029.99±433.89	47293.89±9381.04
P	8104.50±622.62	527.50±14.70	78.39±5.08	2458.48±87.28	69.06±2.23	115.06±1.87	3470.01±672.89
Q	0.00	0.00	51.14±7.26	0.00	0.00	0.00	236.42±3.57
R	10381.43±1166.22	94510.29±923.91	4375.89±638.18	7050.37±427.29	1309.75±78.85	3519.30±377.89	11540.62±1851.31
S	0.00	0.00	0.00	0.00	0.00	0.00	0.00
T	0.00	0.00	0.00	0.00	0.00	0.00	54.50±3.35
U	28812.50±712.67	3049.09±331.80	429.62±57.94	42030.06±2918.32	867.91±121.32	2940.76±429.71	54250.45±514.45
V	6255.68±454.73	0.00	7420.20±1347.81	21154.03±3356.23	34532.17±5472.66	0.00	2497.68±514.49
W	131179.67±23822.30	296360.54±27411.69	177739.67±31325.48	253302.75±13797.93	204966.06±19192.06	112769.37±15304.10	57237.38±8518.95
X	0.00	0.00	0.00	0.00	0.00	0.00	18.17±1.12
Y	63275.73±3149.62	159211.83±22139.00634	42382.50±6880.70	136063.83±9273.34	131293.21±37332.72	106329.75±23582.20	44674.51±4824.84
Z	474705.47±33273.45	431453.40±50807.30	133808.94±22530.40	301043.36±24279.28	303373.70±42881.54	1074298.66±221904.64	363474.24±49626.14
total	24725450.11	34564859.30	5954341.79	18641809.55	6814710.36	32406487.31	6407301.40
