# Supplementary material for: Exploring the Aroma Fingerprint of Various Chinese Pear Cultivars through Qualitative and Quantitative Analysis of Volatile Compounds Using HS-SPME and GC×GC-TOFMS
Source: Molecules. 2023 Jun 15;28(12):4794. doi: 10.3390/molecules28124794 (PMC10301882; doi:10.3390/molecules28124794)
Supplement: Supplementary file 1 [file molecules-28-04794-s001.zip › Table S4.pdf]

Table S4 Primary substances contributing to the grouping

Number	Major contributing substance	M1.p(corr)[1]	M1.p(corr)[2]	Coordinate position
1	Ethyl tiglate	0.362788	0.231943	Frist quadrant
2	(*E*)-2-Hexen-1-ol	0.268774	0.727948	Frist quadrant
3	(*E*)-2-Hexenoic acid, methyl ester	0.249096	0.200189	Frist quadrant
4	Prenyl acetate	0.150338	0.354875	Frist quadrant
5	Methyl 2-ethylacrylate	0.149899	0.354798	Frist quadrant
6	Dimethyl carbonate	0.14983	0.354118	Frist quadrant
7	Ethyl crotonate	0.149492	0.352725	Frist quadrant
8	Methyl isobutyrate	0.149485	0.354454	Frist quadrant
9	(*E*)-2,4-Decadienal	0.14934	0.354802	Frist quadrant
10	Croton aldehyde	0.148406	0.353875	Frist quadrant
11	(E)-1-Methyl-4-(6-methylhept-5-en-2-ylidene)cyclohex-1-ene	0.147482	0.353216	Frist quadrant
12	(*E*)-3-Hexene	0.140929	0.328796	Frist quadrant
13	Methyl decanoate	0.286095	0.518979	Frist quadrant
14	Heptanal	0.274307	0.453346	Frist quadrant
15	3-Methyl-Tridecane	0.269399	0.576113	Frist quadrant
16	2-Methylbutyl acetate	0.175136	0.682395	Frist quadrant
17	Diethyl carbonate	0.145923	0.738236	Frist quadrant
18	(*Z*)-4-Octenoic acid, ethyl ester	-0.924956	0.215929	Third quadrant
19	(*E,Z*)-2,4-Decadienoic acid, ethyl ester	-0.744156	0.193764	Third quadrant
20	(*Z*)-3-Octenoic acid, ethyl ester	-0.947742	0.151031	Third quadrant
21	4-Octenoic acid, methyl ester -	0.782716	0.125507	Third quadrant
22	(*Z*)-4-Decenoic acid, methyl ester	0.937014	0.120803	Third quadrant
23	Ethyl heptanoate	-0.938498	0.103721	Third quadrant

24	Hexyl hexanoate	-0.881531	0.0323526	Third quadrant
25	2-Hexenal	-0.970689	-0.0294891	Third quadrant
26	1-Nonanol	-0.776681	-0.0494806	Third quadrant
27	(*Z,Z*)-*α*-farnesene	-0.836397	-0.0545352	Third quadrant
28	Hexyl caprylate	-0.970932	-0.0606929	Third quadrant
29	2-Methyl propenal	-0.976909	-0.0615247	Third quadrant
30	5-Hexenoic acid, methyl ester	-0.977157	-0.0616024	Third quadrant
31	(*E*)-*β*-ocimene	-0.977484	-0.0616259	Third quadrant
32	Methyl 3-methylthiopropionate	-0.975956	-0.0616632	Third quadrant
33	Hexyl pentanoate	-0.975722	-0.0616714	Third quadrant
34	4-Ethyl-1,2-dimethyl-Benzene	-0.977528	-0.0617072	Third quadrant
35	3-(Methylthio)propanoic acid ethyl ester	-0.976938	-0.0617703	Third quadrant
36	(*Z*)-4-Decenoic acid, ethyl ester	-0.9775	-0.0618425	Third quadrant
37	Hex-5-enoic acid, ethyl ester	-0.977538	-0.061868	Third quadrant
38	2-Methylpropyl hexanoate	-0.976229	-0.0619355	Third quadrant
39	*β*-myrcene	-0.97774	-0.0619785	Third quadrant
40	Butanoic acid, 2-methylpropyl ester	-0.977698	-0.062056	Third quadrant
41	Dodecanoic acid, ethyl ester	-0.976101	-0.0621399	Third quadrant
42	Heptadecane	-0.800251	-0.0974968	Third quadrant
43	Limonene	-0.930021	-0.11968	Third quadrant
44	(*E*)-2-Nonenal	-0.927523	-0.126258	Third quadrant
45	2-Nonanol	-0.896911	-0.130256	Third quadrant
46	(*Z*)-3-Octenoic acid, methyl ester	-0.890955	-0.14788	Third quadrant
47	Propyl hexanoate	-0.976458	-0.148819	Third quadrant
48	2,6,10-Trimethyl-Dodecane	-0.857014	-0.165258	Third quadrant
49	Ethyl propionate	-0.884985	-0.1957	Third quadrant

50	Octyl acetate	-0.878433	-0.202295	Third quadrant
51	Hexyl butyrate	-0.790774	-0.233174	Third quadrant
52	Isobutyl acetate	0.387362	-0.0469614	Third quadrant
53	(*Z*)-Hex-2-enyl acetate	0.379671	-0.28154	Third quadrant
54	Undecane	0.367192	-0.0945826	Third quadrant
55	2-Buten-1-ol, acetate	0.355562	-0.0957354	Fourth quadrant
56	*n*-Heptanol	0.349283	-0.299904	Fourth quadrant
57	1-Octanol	0.347826	-0.352579	Fourth quadrant
58	Methyl (*Z*)-hex-3-enoat	0.329931	e-0.188992	Fourth quadrant
59	3-Methyl-3-buten-1-ol, acetate	0.311441	-0.394704	Fourth quadrant
60	(*Z*)-4-Decenoic acid, methyl ester	0.311315	-0.39465	Fourth quadrant
61	Methyl 2-methyl-2-butenoate	0.30978	-0.393347	Fourth quadrant
62	propyl (*E*)-2-methylbut-2-enoate	0.309408	-0.391215	Fourth quadrant
63	(*Z*)-3-Decen-1-yl acetate	0.307106	-0.390203	Fourth quadrant
64	Heptyl acetate	0.283893	-0.413108	Fourth quadrant
65	Methyl caproate	0.243891	-0.431191	Fourth quadrant
66	2-Methylbut-2-en-1-yl acetate	0.237211	-0.24665	Fourth quadrant
67	Denderalasin	0.237125	-0.246051	Fourth quadrant
68	*o*-Cymene	0.237108	-0.247157	Fourth quadrant
69	Methyl tiglate	0.236964	-0.247067	Fourth quadrant
70	Methyl 3-(methylthio)-(E)-2-propenoate	0.236949	-0.247344	Fourth quadrant
71	Ethyl acetate	0.236754	-0.44709	Fourth quadrant
72	Hexyl tiglate	0.236393	-0.247466	Fourth quadrant
73	1,2,3-Trimethyl-Benzene	0.235831	-0.244298	Fourth quadrant
74	3-Octanol	0.235618	-0.242729	Fourth quadrant
75	Decane	0.23556	-0.246452	Fourth quadrant

76	Propanoic acid, 2-methyl-, 3-hydroxy-2,2,4-trimethylpentyl ester	0.235102	-0.247208	Fourth quadrant
77	2,3-Octanedione	0.23498	-0.242619	Fourth quadrant
78	3-(4-Methylpent-3-enyl)furan	0.23498	-0.243622	Fourth quadrant
79	(*E*)-3-hexenyl acetate	0.233659	-0.0901543	Fourth quadrant
80	Ethyl (*2Z*)-but-2-enoate	0.230993	-0.308653	Fourth quadrant
81	3,7,11-trimethyl-6,10-Dodecadien-1-ol	0.23096	-0.236737	Fourth quadrant
82	1,3,5,7-Cyclooctatetraene	0.213697	-0.0750463	Fourth quadrant
83	Methyl propionate	0.210413	-0.423065	Fourth quadrant
84	Hexyl acetate	0.193771	-0.163106	Fourth quadrant
85	Ethyl caproate	0.166617	-0.0893755	Fourth quadrant
86	Decyl acetate	0.142533	-0.435172	Fourth quadrant
87	(Z)-2-Hexen-1-ol	0.113955	-0.390324	Fourth quadrant
88	4-Hexen-1-ol, acetate	0.113431	-0.38932	Fourth quadrant
89	α-curcumene	0.112893	-0.386183	Fourth quadrant
90	Hexadecane	0.0247137	-0.0742797	Fourth quadrant
91	Ethyl (*Z*)-hex-3-enoate	0.0243329	-0.0739571	Fourth quadrant
92	Isobutyl acetate	0.387362	-0.0469614	Fourth quadrant
93	(*Z*)-hex-2-enyl acetate	0.379671	-0.28154	Fourth quadrant
94	Undecane	0.367192	-0.0945826	Fourth quadrant
